# Dual-faced guardians: SGLT2 inhibitors’ kidney protection and health challenges: a position statement by Kasralainy nephrology group (KANG)

**DOI:** 10.1186/s13098-025-01790-w

**Published:** 2025-06-14

**Authors:** Amin Roshdy Soliman, Mohamed Elkhatib, Sahier El-Khashab, Rasha Ahmed Darwish, Ahmed Fayed, Tarek S. Abdelaziz, Hany Hammad, Rabab Mahmoud Ahmed, Hoda Abdelhamid Maamoun

**Affiliations:** Internal Medicine and Nephrology Department, Faculty of Medicine, Cairo University, Giza, Egypt

**Keywords:** SGLT2 inhibitors, Kidney disease management, Renoprotection

## Abstract

**Background:**

SGLT2 inhibitors represent a revolutionary drug class that delivers benefits exceeding those of diabetes management alone. Initially approved for type 2 diabetes management, research continually demonstrates their protective effects on kidney function across several nephrological conditions, including acute kidney injury (AKI), chronic kidney disease (CKD), dialysis-dependent kidney failure, anemia, metabolic bone disease, polycystic kidney disease (PKD), glomerulonephritis, and kidney transplantation.

**Purpose:**

This study aims to identify how SGLT2 inhibitors modify nephrological care by investigating their mechanisms of action, therapeutic outcomes, and potential applications in multiple kidney diseases. It summarizes clinical trial data alongside mechanistic insights to provide a comprehensive assessment of therapeutic outcomes beyond diabetes mellitus.

**Findings:**

Numerous clinical studies have demonstrated that SGLT2 inhibitors reduce kidney disease progression in patients with or without diabetes. These findings indicate that SGLT2 inhibitors provide kidney protection by enhancing tubuloglomerular feedback, improving renal blood flow, and reducing inflammation and ischemic tissue damage. They also provide cardiovascular benefits to dialysis patients while maintaining effective blood flow during dialysis. SGLT2 inhibitors should not be used in autosomal dominant polycystic kidney disease (ADPKD) outside clinical trials. The potential impact of SGLT2 inhibitors on bone mineral health, particularly regarding bone mineral density (BMD) reduction and fracture risk, requires careful consideration, especially in patients with pre-existing bone health concerns. Kidney transplant recipients benefit from SGLT2 inhibitors’ protective effects on kidney health and assistance with diabetes management; however, further research is needed on drug compatibility with immunosuppressants and infection prevention.

## Introduction

Sodium-glucose cotransporter 2 (SGLT2) inhibitors represent an important advancement in nephrology, offering benefits far beyond glycemic control in individuals with type 2 diabetes mellitus (T2DM). These inhibitors target the SGLT2 transporter in renal proximal tubules, reducing glucose reabsorption and promoting glycosuria [1].

SGLT2 inhibition is a promising new therapy for diabetic kidney disease (DKD). These inhibitors improve glycemic control by enhancing insulin secretion and sensitivity. They also exhibit loop-like diuretic effects and inhibit Na+/H + exchanger NHE3-mediated actions, lowering blood pressure. The reduction of intraglomerular pressure via tubuloglomerular feedback supports their renoprotective role in DKD. Furthermore, they exert anti-inflammatory and antioxidative effects [2].

Dapagliflozin reduced the hazard ratio for a composite renal and cardiovascular death endpoint in patients with chronic kidney disease (CKD) attributed to various causes, with or without type 2 diabetes [3].

Diabetes-induced mitochondrial dysfunction involves abnormal mitophagy, fission, fusion, and biosynthesis. Uncorrected mitochondrial dysfunction accelerates renal decline, worsening DKD. Mitochondrial fusion depends on mitofusin (previously termed “mitofusion”) proteins Mfn1 or Mfn2 and dynamin family GTPase optic atrophy factor 1 (Opa1). Ipragliflozin corrects abnormal Mfn and Opa1 levels without altering blood glucose or body weight. It also decreases transforming growth factor-beta (TGF-β) expression, a key cytokine in renal apoptosis and DKD progression. Empagliflozin reduces diabetes-induced mitochondrial reactive oxygen species (ROS), which correlate with impaired mitophagy [4].

The glucose-lowering effects of SGLT2 inhibitors are complemented by significant nephroprotective mechanisms:

1. reducing intraglomerular pressure by modulating renal hemodynamics, thereby alleviating stress on the glomeruli;
2. improving tubuloglomerular feedback by restoring the feedback loop, enhancing sodium handling and reducing sodium reabsorption; and.
3. reducing albuminuria by mitigating glomerular damage, which decreases proteinuria and preserves kidney function [5].

Additionally, SGLT2 inhibitors exhibit cardiovascular benefits, including reduced heart failure risk and improved outcomes in patients with preserved or reduced ejection fraction. Expanding evidence supports their use in diabetic and nondiabetic CKD, heart failure, and potentially type 1 diabetes [6, 7].

Current indications for SGLT2 inhibitors include CKD, diabetic kidney disease (DKD), heart failure with reduced ejection fraction (HFrEF), and heart failure with preserved ejection fraction (HFpEF). Emerging evidence indicates these drugs are also prescribed to patients with type 1 diabetes mellitus. Approved SGLT2 inhibitors include canagliflozin, dapagliflozin, empagliflozin, ertugliflozin, and sotagliflozin. The latter is the first dual SGLT1 and SGLT2 inhibitor and is approved in Europe for both type 1 diabetes mellitus and T2DM [8].

As a result, SGLT2 inhibitors have become indispensable in managing CKD, DKD, and associated cardiovascular risks, underscoring their growing importance in nephrology. However, careful patient selection, monitoring, and management of potential side effects are essential.

Review Points.

- Verify the latest clinical guidelines regarding the use of SGLT2 inhibitors in nephrology.
- Include recent trial data and ensure that the draft incorporates the most current evidence.
- Ensure accuracy in recommended dose adjustments and monitoring protocols for patients with varying levels of kidney function.

## Methods

### *Objectives* and scope

**Fig. 1:**
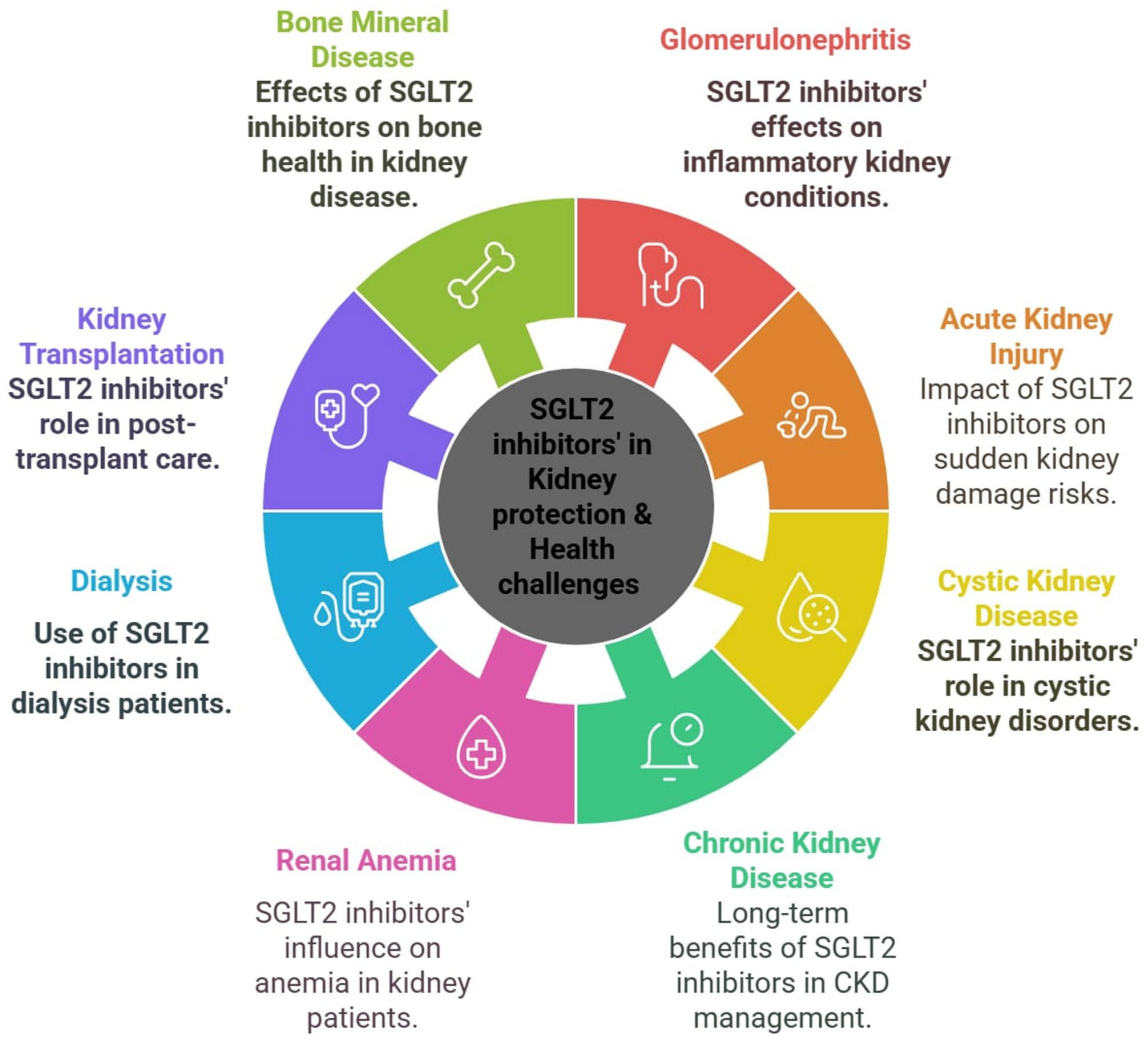
SGLT2 Inhibitors’ Kidney Protection and Health Challenges

The primary objective of this review is to determine the effectiveness of SGLT2 inhibitors in different kidney diseases, including chronic kidney disease, acute kidney injury, polycystic kidney disease, renal anemia, glomerulonephritis, bone mineral disease, dialysis, and transplantation. This review aims to compare clinical efficacy, modes of action, and safety for each condition; review outcomes, actions, and risks in each disease; and analyze the current status and potential future applications for each indication (Fig. 1).

### Literature search strategy

This review employs a systematic narrative design, combining appraisal of search strategies with thematic synthesis. Literature searches were conducted in PubMed, Embase, the Cochrane Library, Scopus, and Web of Science.

### Search keywords

Combined keywords and Medical Subject Headings (MeSH) terms for each condition:

- General Terms: “SGLT2 inhibitors,” “nephrology”.
- CKD: “chronic kidney disease,” “renal protection,” “proteinuria,” “eGFR”.
- AKI: “acute kidney injury,” “renal ischemia,” and “nephrotoxicity”.
- PKD: “Polycystic kidney disease,” “autosomal dominant PKD,” and “renal cysts”.
- Renal anemia: “anemia,” “erythropoiesis,” “erythropoietin”.
- Glomerulonephritis: “IgA nephropathy,” “glomerular disease,” and “proteinuria”.
- Bone mineral disease: “bone health,” “osteoporosis,” “phosphate metabolism”.
- Dialysis: “hemodialysis,” “hemodialysis,” and “peritoneal dialysis”.
- Transplantation: “kidney transplant,” “allograft function,” and “posttransplant outcomes”.

Inclusion criteria: Articles from peer-reviewed journals (randomized controlled trials, cohort studies, systematic reviews, meta-analyses); articles indexed in English and published up to 2025 and research conducted with renal endpoints, risk or efficacy profiles, or active pharmaceutical ingredients.

### Data extraction

Using design templates to store important information:

- Study details: Author, year, journal, study design, and population of the reviewed papers.
- Interventions: Dosage, duration, and groups with and without comparators.
- Outcomes: changes in eGFR, proteinuria, cardiovascular safety, and tolerability.
- Mechanisms: the renal and extrarenal pathways.
- Relevance to conditions: general outcomes/complications in CKD, AKI, PKD, DKD, etc.

### Screening process

Initial Screening: Titles and abstracts were reviewed to determine relevance based on pre-defined criteria.

Full-Text Review: Articles passing the initial screening were assessed in full text for quality and relevance.

### Quality assessment

The quality of the included studies was evaluated via the appropriate tools:

- Randomized Controlled Trials: Cochrane Risk of Bias Tool.
- Case‒Control/Cross-Sectional/Observational Trials: Newcastle–Ottawa Scale.
- Systematic reviews: AMSTAR checklist, GRADE system for evidence quality.

### Data synthesis

The data were analyzed via thematic analysis according to renal condition:

- CKD: Specific objectives for renal and cardiovascular outcomes are summarized.
- AKI: Analysis of possible factors that can minimize or aggravate the risk of AKI and/or AKI recovery.
- PKD: Data on cyst growth control.
- Renal anemia: Assessment of erythropoietin activity and possible combination with ESA.
- Glomerulonephritis: Analysis of disease type effects, e.g., IgA nephropathy and lupus nephritis.
- Bone mineral disease: Recommendations and possible effects on bone density and fractures.
- Dialysis: Volume control, interdialytic weight, and cardiovascular outcomes.
- Transplantation: Data on graft function, rejection risk, and safety.

### Research questions

- General: How do SGLT2 inhibitors affect renal function and prognosis in patients with various nephrological disorders?
- CKD: What is the impact of SGLT2 inhibitors on renal and cardiovascular outcomes in patients with CKD?
- AKI: Do SGLT2 inhibitors prevent or worsen AKI outcomes?
- PKD: Is there a clinical advantage in reducing cyst progression or maintaining renal function in patients with PKD?
- Renal anemia: Do SGLT2 inhibitors affect erythropoiesis or iron metabolism?
- Glomerulonephritis: What evidence supports the benefits of SGLT2 inhibitors in treating patients with glomerular diseases such as IgA nephropathy?
- Bone mineral disease: What are the effects of SGLT2 inhibitors on bone and bone mineral metabolism?
- Dialysis: What are the benefits of SGLT2 inhibitors in patients undergoing dialysis?
- Transplantation: Are SGLT2 inhibitors safe and effective in kidney transplant recipients?

## SGLT2 inhibitors and chronic kidney disease (CKD)

Chronic kidney disease (CKD) affects millions of people worldwide, increasing cardiovascular risk and progressing to end-stage kidney disease (ESKD). Sodium-glucose cotransporter 2 inhibitors (SGLT2is) have shown promise in CKD treatment, irrespective of type 2 diabetes (T2D) status, offering both renal and cardiovascular benefits. They prevent a decline in kidney function independently of their effect on glycemic control [9, 10].

In diabetic kidney disease (DKD), SGLT2 inhibitors slow progression by improving glycemic control through increased insulin sensitivity and glucose excretion, reducing albuminuria, and slowing the decline in estimated glomerular filtration rate (eGFR). In CKD patients without diabetes mellitus, SGLT2 inhibitors provide renal protection via mechanisms beyond reducing eGFR decline and antiproteinuric effects. These mechanisms include increased uric acid excretion, decreased serum uric acid, inhibition of sodium-hydrogen exchangers, reduced oxidative stress, anti-inflammatory effects, promotion of autophagy, reversal of renal hypoxia, enhanced ketogenesis, and prevention of aberrant glycolysis and renal senescence [11].

Regarding combination therapy with SGLT2 inhibitors and other agents such as mineralocorticoid receptor antagonists (MRAs) and glucagon-like peptide-1 receptor agonists (GLP-1RAs): SGLT2 inhibitors have demonstrated significant and clinically relevant reductions in albuminuria, nephropathy progression, doubling of serum creatinine levels, and initiation of renal replacement therapy. Additionally, incretin-based agents like liraglutide (a GLP-1 analog) and linagliptin (a dipeptidyl peptidase-4 inhibitor) exhibit vasotropic effects, suggesting potential to reduce the risk of DKD [12].

Until recently, MRAs were unsuitable for DKD treatment due to hyperkalemia risk. However, finerenone, a non-steroidal MRA, significantly reduced renal composite endpoints without causing severe hyperkalemia necessitating discontinuation (FIDELIO-DKD trial). Thus, the mainstay treatments for DKD now include renin-angiotensin system (RAS) inhibitors, SGLT2 inhibitors, incretin-based agents, and non-steroidal MRAs—collectively referred to as the DKD “fantastic four” [12].

### SGLT2 inhibitors in different CKD stages

#### Stage 3 CKD

In stage 3 CKD, SGLT2 inhibitors reduce the risk of primary cardiovascular outcomes by 26% overall: 30% in stage 3a, 23% in stage 3b, and 29% in stage 4 CKD. Dapagliflozin also favorably influences cholesterol fractions in stage 3 CKD patients, decreasing total cholesterol, LDL-C, triglycerides, serum creatinine, and the albumin–creatinine ratio, while increasing HDL-C and eGFR [13].

#### Stage 4 CKD

The DAPA-CKD study emphasized dapagliflozin’s renoprotective effects in CKD patients with eGFR between 25 and 30 ml/min/1.73 m². Regardless of diabetes status, it remains debatable whether SGLT2 inhibitors should be continued in this group. Although evidence for kidney-related endpoints is limited for eGFR < 25, some researchers suggest continuing SGLT2 inhibitors until dialysis initiation. Others recommend against initiating SGLT2 inhibitors at very low GFR due to an initial dip in GFR and advise discontinuation if acute kidney injury (AKI), hypovolemia, or hypotension occurs [14].

#### CKD with and without albuminuria

Previously, it was believed that only CKD patients with proteinuria benefit from SGLT2 inhibitors. However, a meta-analysis of cardiovascular outcome trials (CVOTs) revealed that SGLT2 inhibitors delay CKD progression regardless of baseline albuminuria. The EMPA-KIDNEY trial, stopped early in March 2022 for efficacy, suggested that CKD patients without albuminuria may also benefit from SGLT2 inhibitors [15].

### SGLT2 inhibitors in elderly individuals and children with CKD

Geriatric populations (≥ 75 years) have been underrepresented in clinical trials evaluating the renoprotective effects of SGLT2 inhibitors, resulting in limited data on their efficacy in elderly DKD patients. It remains unclear whether renal benefits differ between patients with or without proteinuria [16, 17].

Until recently, SGLT2 inhibitors were approved only for adults and cats, not for children with CKD. Bexagliflozin was approved for use in all mammals, including cats, opening a market in the USA alone of over 100,000 overweight cats. It received the Pet Innovation Award “Overall Cat Health Product of the Year 2023.” However, it has not been tested in children with CKD in Europe or the USA [18]. The FDA approved empagliflozin for treating T2DM in children aged 10 years and older.

## Conclusion

Recent studies confirm that SGLT2 inhibitors offer significant benefits for CKD patients with and without diabetes mellitus, including those with or without heart failure. They improve renal outcomes, reduce cardiovascular risk, and aid glycemic control. These agents should be used with caution, monitored regularly, and withheld if complications arise. Careful patient selection, dose titration, early detection of adverse effects, and close follow-up are essential.

### Recommendations by Kasralainy nephrology group (KANG)

- Patient selection: SGLT2 inhibitors should be prescribed cautiously, especially in elderly individuals and those with atherosclerotic cardiovascular disease or peripheral vascular disease.
- Patient education: Patients should be informed about possible complications. Routine foot care is recommended, particularly for those with diabetes. Maintaining genital hygiene is important. Precautions should be taken during sick days and fasting periods (e.g., Ramadan). A ketogenic diet is not advised.
- Monitoring: Regular monitoring of vital signs and renal function is crucial.
- Dosing: It is preferable to start with low doses (e.g., empagliflozin 10 mg) and adjust based on tolerance.
- Medication adjustments: Other medications, including diuretics, hypoglycemic agents, and antihypertensives, should be reduced as appropriate.

Our recommendations are based on high-quality evidence, including secondary analyses from the DAPA-CKD and EMPA-KIDNEY trials, as well as real-world observational studies. Observational data also highlight potential controversial effects of SGLT2 inhibitors in elderly patients, those with decompensated heart failure, and patients with macrovascular complications.

## SGLT2 inhibitors and dialysis

Many health organizations recommend discontinuing SGLT2 inhibitors (SGLT2is) in patients with severe renal impairment; however, clinical trials have been conducted in patients receiving dialysis. The recommendation is to consider SGLT2is in patients with heart failure with reduced ejection fraction (HFrEF) and hospitalized heart failure patients if the estimated glomerular filtration rate (eGFR) is greater than 20 ml/min/1.73 m². There are no contraindications for low-dose use: the SGLT2 inhibitors dapagliflozin and empagliflozin should not exceed 10 mg/day, and canagliflozin should not exceed 25 mg/day. Recent guidelines do not advocate initiating SGLT2is in patients receiving dialysis with heart failure and recommend discontinuation if the patient is off dialysis [19].

A recent trial involving 125 patients with eGFRs below 30 ml/min/1.73 m² and stage 5 dialysis (G5D) demonstrated the effectiveness of dapagliflozin and the non-adenosine nature of the treatment. The data provide solid evidence supporting the use of SGLT2is in patients on dialysis. Three phase III randomized controlled trials (RCTs) investigating SGLT2is, aimed at understanding their impact on kidney survival, cardiovascular events, and mortality, were reviewed to assess benefits for patient management. The nephrology society has modified recommendations for patients with a GFR below 30 ml/min/1.73 m² regarding high doses of the tested SGLT2is. Multidisciplinary stewardship should be applied when using SGLT2is in G5D patients due to patient preferences, the presence of hyperkalemia, fluid restrictions, and comorbidities. Experts advise administering SGLT2is at least 2 h post-dialysis and adjusting doses based on weekly drug clearance [20].

### Benefits and harms of SGLT2 inhibitors in patients on dialysis

Studies have examined the benefits and side effects of SGLT2 inhibitors in adult patients with end-stage renal disease (ESRD) receiving renal replacement therapy. These drugs help lower HbA1c in hyperglycemic patients and support better glucose levels in the urine. Benefits may include appetite regulation, control of obesity, hypertension, and cardiovascular diseases; improved insulin sensitivity; and lowered LDL cholesterol and uric acid. Expected outcomes include enhanced dialysis quality, reduced interdialytic weight gain, decreased peritoneal refilling in peritoneal dialysis, and improvements in neuropathy, diabetic foot ulcers, quality of life, and sexual function [21].

Specific concerns in the dialysis population include over-restrictive calorie intake and nonadherence to energy requirements, especially in hemodialysis patients who require more carbohydrates. SGLT2 inhibitors can critically exacerbate severe heart failure and cerebrovascular accidents due to osmotic diuresis. In hemodialysis, side effects may include increased dry weight and overhydration. Some complications of peritoneal dialysis may affect catheter patency, increase local effects on host defense, and alter intraperitoneal pressure. Moreover, achieving optimal metabolic and electrolyte balance presents unique dialysis-related challenges, including glucose control and replenishment of glucose losses during dialysis [22].

Several important points must be considered when using SGLT2 inhibitors in dialysis patients [23–25]:

- Limited evidence makes it difficult to assess the usefulness of SGLT2 inhibitors in ESRD patients receiving dialysis because their effectiveness diminishes as GFR falls below 45 ml/min.
- Differences in dialysis effectiveness exist between hemodialysis and peritoneal dialysis systems; these drugs remain longer in peritoneal dialysis systems than in hemodialysis, complicating dosing schedules (see Table 1).
- SGLT2 inhibitor use increases the risk of hypoglycemia and poses safety risks related to volume management and urinary and genital infections, particularly in immunocompromised patients.
- Evidence shows that residual kidney function remains mostly unaffected by these medications; however, research on their effects in dialysis patients is scarce.
- Limited evidence exists for SGLT2 inhibitor use in dialysis patients because research on dialysis populations is scarce; nonetheless, results from non-dialysis studies form the basis for recommending their use.
- Long-term management of dialysis patients must rely on real-time patient oversight, as individualized care strategies show promise for better clinical outcomes.

The use of SGLT2 inhibitors in chronic kidney disease (CKD) patients receiving dialysis requires careful evaluation, as more research and specialized patient care systems are needed.

**Table 1:** Difference between Hemodialysis and peritoneal dialysis regarding the use of SGLT2 inhibitors

Aspect	Hemodialysis	Peritoneal dialysis
**Pharmacokinetics**	SGLT2 inhibitors may be excreted during dialysis due to their removal by hemodialysis. This can result in lower plasma concentrations of the drug.	SGLT2 inhibitors may be less affected due to slower absorption; however, metabolic clearance can occur.
**Excretion and dosing**	Administer SGLT2 inhibitors after dialysis sessions to maintain effective drug levels.	Administration may include considerations for dwell time and drug retention in the peritoneal cavity.
**Efficacy in blood glucose control**	May have reduced efficacy due to the removal of glucose and the drug during dialysis.	Potentially better maintenance of glucose control as some residual renal function may be preserved.
**Impact on residual kidney function (RKF)**	Higher likelihood of SGLT2 inhibitors protecting RKF in patients with residual function.	Use may help preserve RKF, which is crucial for patient outcomes in peritoneal dialysis.
**Guidelines and recommendations**	Current guidelines lack specific recommendations for SGLT2 inhibitor use in hemodialysis; caution is advised due to eGFR fluctuations.	Recommendations advocate studying the benefits and safety of SGLT2 inhibitors in this population.
**Clinical evidence**	Limited studies specifically address their use in hemodialysis patients.	Few studies exist; however, emerging evidence indicates potential safety and efficacy concerns.

### Recommendations by Kasralainy nephrology group (KANG)

- Clinicians should employ justified patient stratification and glycemic management to achieve non-cardiovascular benefits beyond avoiding SGLT2 inhibitor-associated amputation. This includes monitoring electrolytes in patients on dialysis and those with higher cardiovascular risk, as well as monitoring bone indices.
- Patients with end-stage renal disease (ESRD) should not use SGLT2 inhibitors without adequate, proper, close, individualized supervision and monitoring.

## SGLT2 inhibitors and acute kidney injury (AKI)

Acute kidney injury (AKI) is a major risk factor for developing chronic kidney disease (CKD). Patients with diabetes mellitus are particularly susceptible to recurrent AKI episodes, which can accelerate the progression to end-stage kidney disease (ESKD) [14].

As explained earlier, SGLT2 inhibitors have nephroprotective effects on the progression of diabetic kidney disease as well as nondiabetic chronic kidney disease [26]. However, the underlying pathophysiologic mechanisms are not yet fully understood.

Following reported cases of AKI in the U.S. Food and Drug Administration Adverse Event Reporting System (FDAERS), there has been significant interest in analyzing the true risk of AKI associated with SGLT2 inhibitors. Several propensity score-matched analyses have revealed a reduction in AKI risk with the use of SGLT2 inhibitors.

Subsequent sensitivity analyses, adjusting for confounders such as age, sex, and ACE inhibitor use, have confirmed that SGLT2 inhibitors are associated with a lower risk of AKI (i.e., they are kidney-safe) [27]. Some studies have shown that SGLT2 inhibitor use may be linked to a decreased risk of developing AKI [28].

One large report from Taiwan analyzed data from more than 104,000 patients and found that, after a median follow-up of 2.5 years, patients using SGLT2 inhibitors had a lower incidence of AKI compared to those using DPP-4 inhibitors.

A meta-analysis pooling data from 38,723 patients across four major clinical trials also reported a lower incidence of AKI in patients with diabetes [29]. Furthermore, a systematic review and meta-analysis including 112 randomized controlled trials (totaling over 92,000 patients) and four observational studies (more than 83,900 patients) demonstrated that SGLT2 inhibitors reduce the occurrence of AKI by 36% [30].

SGLT2 inhibitors reduce glomerular pressure, causing an initial decrease in the glomerular filtration rate (GFR), an effect similar to that of ACE inhibitors and ARBs. However, this reduction in glomerular pressure is renoprotective, as it mitigates podocyte stress [31]. Additionally, SGLT2 inhibitors significantly reduce major adverse cardiovascular events in patients with diabetes and in those with heart failure without diabetes. These favorable cardiac effects may reduce the incidence of cardiorenal syndrome.

Interestingly, previous clinical trials showed that a single 50 mg dose of dapagliflozin improved renal cortical oxygenation, as observed via MRI of the kidney [32]. This effect was independent of renal perfusion or blood flow [33].

Other favorable renal effects include reducing inflammation. SGLT2 inhibitors may mitigate sepsis, a major cause of AKI [34]. They also show promise in preventing contrast-induced nephropathy [35]. A recent meta-analysis synthesizing evidence from 2,572 patients undergoing coronary angiography or percutaneous coronary intervention (PCI) found that dapagliflozin reduces the incidence of contrast-induced nephropathy by up to 63% [36].

Studies have demonstrated the benefit of dapagliflozin in reducing the risk of AKI in patients with acute myocardial infarction undergoing PCI [37].

### Recommendations by the Kasralainy nephrology group (KANG)

- Patients with Chronic Kidney Disease on SGLT2 Inhibitors:
- Patients with chronic kidney disease on long-term SGLT2 inhibitors are advised to withhold these medications during acute illnesses, especially diarrheal illness, to reduce the risk of hypovolemia. Although guidelines recommend this, strong evidence supporting this practice is lacking [38]. The decision to restart SGLT2 inhibitors should be delayed until renal function improves and the risk of further kidney injury diminishes.
- Prevention of Contrast-Induced Nephropathy:
- SGLT2 inhibitors may be used to prevent contrast-induced nephropathy, based on limited evidence. This recommendation is supported by a recent meta-analysis [36].
- Continuation of SGLT2 Inhibitors After Acute Myocardial Infarction:
- We recommend continuing SGLT2 inhibitors to prevent AKI after acute myocardial infarction in patients undergoing primary coronary intervention (PCI). This recommendation is based on weak evidence from a retrospective study [37].
- Use of SGLT2 Inhibitors in Patients with AKI:
- The decision to use SGLT2 inhibitors in patients with AKI should be made on a case-by-case basis, considering the severity of kidney injury, the presence of underlying kidney disease, and the overall clinical status of the patient.

## SGLT2 inhibitors and bone mineral disease

SGLT2 inhibitors are a valuable class of drugs for managing type 2 diabetes and have demonstrated positive effects on cardiovascular and renal health. However, their potential impact on bone mineral health, particularly regarding bone mineral density (BMD) reduction and fracture risk, requires careful consideration, especially in patients with preexisting bone health concerns [39].

SGLT2 inhibitors increase urinary calcium excretion, which may contribute to a reduction in circulating calcium levels. This could stimulate compensatory mechanisms that affect bone health. Changes in calcium and phosphate homeostasis could influence the body’s ability to maintain healthy bone mineralization, although further research is needed to clarify these effects [40].

SGLT2 inhibitors can alter bone remodeling processes. For example, they may increase osteoclast activity and affect the balance between bone formation and resorption. Other studies have suggested that SGLT2 inhibitors might increase osteoblast activity, potentially counteracting the negative effects of bone resorption, although this effect has not been conclusively proven in clinical practice [41].

Some studies suggest that SGLT2 inhibitors may lead to slight decreases in bone mineral density, particularly in certain populations such as older adults or those with preexisting bone health issues. The mechanism behind this potential increase in bone fragility is not fully understood but may be related to changes in calcium and phosphate metabolism [42].

A study published in the Journal of Bone and Mineral Research reported that SGLT2 inhibitors were associated with a small but significant reduction in bone mineral density over time, especially in postmenopausal women and individuals with a history of fractures [43].

On the other hand, the EMPA-REG OUTCOME trial, which examined empagliflozin (an SGLT2 inhibitor), did not show any significant increase in fracture risk, indicating that the effects on bone health may vary between individuals and depend on factors such as age, sex, and preexisting bone conditions [44].

The American Diabetes Association (ADA) has noted concerns regarding the potential impact of SGLT2 inhibitors on bone health. Specifically, the CANVAS study indicated a higher proportion of fractures in the canagliflozin group compared to the placebo group (2.7% vs. 1.9%). However, subsequent analyses from the CANVAS and CREDENCE trials suggested a neutral effect on fracture risk [45, 46].

Meta-analyses have provided further insights. Wang et al. found no significant association between SGLT2 inhibitor use and increased fracture risk (pooled relative risk = 1.21, 95% confidence interval [0.95, 1.54]) and no substantial effects on bone mineral density at various skeletal sites [47]. Similarly, Li et al. reported no increased risk of fractures and no significant impact on BMD in patients treated with SGLT2 inhibitors [48].

In patients with chronic kidney disease (CKD), SGLT2 inhibitors have been associated with alterations in bone and mineral metabolism, including increased serum phosphate, fibroblast growth factor-23 (FGF-23), and parathyroid hormone (PTH) levels, along with decreased 1,25-dihydroxyvitamin D levels. Despite these changes, clinical trials have consistently demonstrated no increased fracture risk in this population [49].

In summary, while some concerns exist about the potential impact of SGLT2 inhibitors on bone health, the majority of clinical evidence suggests a neutral effect on fracture risk and BMD. However, careful monitoring and further research are warranted, especially in populations at higher risk for bone disease.

### Recommendations by Kasralainy nephrology group (KANG)

- In patients receiving SGLT2 inhibitors, ensuring adequate calcium and vitamin D intake to support bone health may be beneficial. Additionally, regular assessment of BMD may be considered in those with other risk factors for bone disease.
- Ongoing long-term follow-up studies are crucial for determining the safety of SGLT2 inhibitors in relation to bone health. The overall impact may depend on individual patient risk factors.

### SGLT2 inhibitors and renal anemia

Since the first clinical trials in patients with type 2 diabetes mellitus, treatment with SGLT2 inhibitors (SGLT2i) has produced an early and sustained increase in hemoglobin and hematocrit levels of 0.5–0.7 g/dL, a finding subsequently confirmed in clinical trials involving patients with heart failure (HF) and chronic kidney disease (CKD) [50–52].

These changes were initially attributed to hemoconcentration due to the diuretic effect and decreased plasma volume [53]. However, it was soon suggested that other mechanisms might be involved, given that the increase exceeded what would be expected from initial plasma volume contraction alone [54].

Among the multifactorial mechanisms proposed is the role of SGLT2i in improving both erythropoiesis and iron metabolism [55]. The increase in erythropoiesis may result from the reversal of relative tissue hypoxia in the proximal tubule, caused by decreased activity of the Na+/K + ATPase pump secondary to reduced sodium reabsorption induced by SGLT2i. The normalization of renal cortical oxygenation reduces metabolic stress and improves tubulointerstitial function, restoring erythropoietin (EPO) production by peritubular fibroblasts and stimulating erythropoiesis [56]. EPO production, erythropoiesis, and the response to EPO by bone marrow erythroblasts are also enhanced by the anti-inflammatory effects of SGLT2i. Another EPO-independent mechanism identified in experimental studies [57] is the stimulation of vasopressin synthesis by SGLT2i, as this hormone has been shown to increase red blood cell count [58].

SGLT2i also affect iron metabolism, improving functional iron deficiency and increasing intracellular iron availability and utilization, which favors erythropoiesis and myocardial metabolic efficiency [59]. Several studies have reported decreases in hepcidin levels, increases in total iron-binding capacity and soluble transferrin receptor, decreases in transferrin saturation index, and increased expression of transferrin receptor types 1 and 2 in mononuclear cells [14, 60].

This effect of SGLT2i on hemoglobin normalization is expected to reduce the risk of cardiovascular events, including HF and renal events, in patients with CKD. Studies have shown that in patients with HF, an increase in hemoglobin with SGLT2i is closely associated with a reduced risk of adverse events [61] and a better prognosis compared to patients with persistent anemia. In the DAPA-CKD study, the benefit of dapagliflozin on renal events was greater in anemic subjects than in non-anemic subjects. Functional improvements included enhanced quality of life and decreased natriuretic peptide levels in patients with HF [62].

However, it is unlikely that the cardiovascular and renal benefits of SGLT2i are solely attributable to increased hemoglobin and hematocrit. This conclusion is supported by previous studies with erythropoiesis-stimulating agents (ESAs), including hypoxia-inducible factor prolyl-hydroxylase inhibitors (HIF-PHIs), which failed to demonstrate cardiovascular or renal benefits from hemoglobin normalization despite improving ferrokinetics [63, 64].

These findings suggest that the clinical benefits observed with SGLT2i treatment and their association with increased hemoglobin and hematocrit result from multiple complex pathophysiological mechanisms beyond the direct effect of increased hemoglobin.

A potential concern regarding the hemoglobin increase with SGLT2i is a possible association with thrombotic events, as observed in the Reduction of Events by Darbepoetin Alfa in Heart Failure (RED-HF) study following darbepoetin treatment [65]. Fortunately, this has not been observed with SGLT2i, except in cases involving subsequent intravenous (IV) iron administration, which may increase blood viscosity and the risk of cardiovascular events [66]. Therefore, further studies are needed to assess the efficacy and safety of IV iron administration in patients with heart failure and iron deficiency treated with SGLT2i.

Since moderate to severe anemia typically develops from CKD stage IIIb–IV onward, and most patients enrolled in randomized clinical trials testing SGLT2 inhibitors were not in stage IV or V CKD, there is limited information on the potential benefits of SGLT2 inhibitors in severe anemia. Accordingly, they should be used cautiously in patients with very low hemoglobin [67].

Moreover, current data suggest a prognostic role for SGLT2 inhibitors. They may provide beneficial “anti-anemic” effects and delay or prevent the initiation of anemia therapy, including iron supplementation and erythropoiesis-stimulating agents [68]. Conversely, excessive hemoglobin increases should be avoided when adding SGLT2 inhibitors to patients already treated with ESAs and having hemoglobin levels within the optimal target range of 10–12 g/dL, as recommended by the European Renal Best Practice. Caution is warranted due to concerns about a potential increased risk of thrombotic events [69].

In conclusion, the increase in hemoglobin and hematocrit is partially independent of the patient’s anemic status. Therefore, raising hemoglobin levels should not be automatically viewed as beneficial but rather as a double-edged sword—potentially advantageous in anemic patients but potentially harmful in non-anemic individuals, especially those with hemoglobin levels at the upper limit of normal at treatment initiation.

### Recommendations by Kasralainy nephrology group (KANG)

- SGLT2 inhibitors are increasingly recommended for treating patients with chronic kidney disease (CKD) and anemia. These medications are associated with improved cardiorenal outcomes, reduced primary renal events, and enhanced quality of life.
- SGLT2 inhibitors have a prognostic role with “anti-anemic” effects, delaying or preventing the need for anemia treatments, including iron supplementation and erythropoiesis-stimulating agents.
- SGLT2 inhibitors should be used cautiously in non-anemic patients, particularly those with hemoglobin levels at the upper limits of normal at treatment initiation, due to concerns about an increased risk of thrombotic events. Similarly, subsequent IV iron administration may induce hypercoagulability, increasing blood viscosity and cardiovascular risk.
- SGLT2 inhibitors should also be used cautiously in patients with severe anemia, which typically develops in later CKD stages. Since their use is contraindicated in such cases, data on potential benefits in severe anemia are limited.

## SGLT2 inhibitors and glomerulonephritis

SGLT2 inhibitors (SGLT2is) are prescribed for glomerular diseases in combination with renin-angiotensin system (RAS) blockade to enhance proteinuria reduction in patients who have not achieved complete remission with RAS blockade alone. The application of SGLT2 inhibitors in immune-mediated renal diseases is steadily advancing owing to their proven anti-inflammatory and immunomodulatory properties [70].

It is currently not possible to generalize the benefits of SGLT2is for all patients with glomerular disease in terms of proteinuria reduction, which is the main therapeutic target, as this outcome is influenced by a patient’s clinical profile. Patients with serum albumin levels ≥ 3.5 g/dL and a higher body mass index are more likely to benefit from SGLT2is for proteinuria reduction, according to a recent study. Conversely, patients with active disease, characterized by serum albumin levels < 3.5 g/dL or nephrotic-range proteinuria (NRP), are less likely to respond (26%) to SGLT2is for proteinuria reduction than patients with subnephrotic-range proteinuria (74%), although NRP patients may still benefit from SGLT2is in terms of kidney disease protection [70].

### Conflict

ing results have been reported between studies regarding the effect of glomerular disease etiology on the response to SGLT2is for proteinuria reduction. This is due to the retrospective design of these studies, the lack of controls, and the diverse nature of the glomerular diseases involved, which limits the strength of their conclusions. Generally, no significant difference was found concerning a patient’s diabetes background. Some studies have reported that SGLT2i treatment has no significant effect on proteinuria in IgA nephropathy and focal segmental glomerulosclerosis (FSGS) patients [71]. In a recent real-world clinical study, patients with immune complex-mediated membranoproliferative glomerulonephritis and C3 glomerulopathy presented the greatest reduction in proteinuria among various types of primary and secondary glomerular diseases, followed by those with IgA nephropathy and lupus nephritis. Patients with minimal change disease and FSGS were the least responsive [70].

Notably, current studies do not conclusively determine whether the observed reductions in proteinuria result in complete or partial remission. In patients with nondiabetic glomerular disorders, more research is needed to examine SGLT2 expression and its impact on mesangial cells and podocytes to accurately evaluate how SGLT2is affect hematuria in glomerulonephritis patients.

While no improvement in disease activity index scores was observed in lupus nephritis patients, SGLT2is may serve as adjuvant immunomodulatory therapy for systemic lupus erythematosus (SLE), helping to stabilize or increase complement levels overall [72].

Despite the availability of three subtypes of SGLT2 inhibitors—dapagliflozin 10 mg/day, empagliflozin 10 mg/day, and canagliflozin 100 mg/day—the majority of study participants primarily used dapagliflozin. This could affect the applicability of the results to the full class of SGLT2is [70].

When receiving immunosuppressive therapy for glomerular disorders, SGLT2is are considered safe. However, relapse of the underlying glomerular illness and a lack of antiproteinuric effectiveness are hazards, albeit rare. Patients with lupus may also be at increased risk for osteoporosis, vascular issues, and infections [70, 72].

SGLT2 inhibitors have been shown in most studies to slow kidney disease progression, offer cardiovascular advantages, and lower blood pressure. However, there have been conflicting findings regarding these results in glomerular disease, including lupus nephritis. More validation with large sample sizes is necessary to ascertain whether there is a substantial difference compared with patients not receiving SGLT2i treatment [71].

Lipids have been linked to SGLT2 inhibitors. There is no research on how SGLT2 inhibitors affect lipid profiles in nondiabetic glomerulonephritis patients. Dapagliflozin has a positive effect on the lipid profile in nondiabetic CKD patients, according to a recent study [73].

### Recommendations by Kasralainy nephrology group (KANG)

When considering the use of SGLT2 inhibitors (SGLT2is) for patients with glomerular disease, it is essential to evaluate several key factors that can significantly impact treatment efficacy in terms of proteinuria reduction. These include:

- The etiology and immune background of the glomerular disease.
- The chronicity of the disease as observed in kidney biopsies.
- The patient’s history of diabetes mellitus and the specific type of SGLT2i used.
- Whether the patient is receiving concomitant immunosuppressive therapy.
- Baseline serum creatinine and serum albumin levels and the degree of proteinuria.

These factors collectively influence treatment outcomes and should guide clinical decision-making to optimize the potential benefits of SGLT2is in glomerular disease patients.

Our recommendations are founded on high-quality evidence, including secondary subanalyses from the DAPA-CKD and EMPA-KIDNEY trials, as well as data from real-world observational clinical studies. Additionally, the potential nephroprotective effects of SGLT2 inhibitors in systemic lupus erythematosus (SLE) are supported by preliminary findings from animal models and small-scale observational studies.

## SGLT2 inhibitors and kidney transplantation

The well-established benefits of SGLT2 inhibitors (SGLT2is) in nontransplant populations have sparked interest in their potential applications for kidney transplant recipients (KTRs), who face unique metabolic and cardiovascular challenges, including posttransplant diabetes mellitus (PTDM), obesity, and chronic allograft dysfunction. These conditions significantly influence patient morbidity, mortality, and allograft survival. SGLT2is have demonstrated short-term efficacy, impacting HbA1c, estimated glomerular filtration rate (eGFR), hemoglobin/hematocrit, serum uric acid, and serum magnesium levels. They have also shown a favorable safety profile in KTRs, with low rates of infections, hypoglycemia, euglycemic diabetic ketoacidosis, and stable tacrolimus levels [74]. However, the application of SGLT2is in kidney transplantation is largely based on observational studies and small randomized controlled trials (RCTs), with encouraging data, but long-term outcomes remain uncertain. The available evidence on the safety and efficacy of SGLT2i therapy in KTRs is very limited, encompassing only nine published studies as of 2021, consisting of eight manuscripts and one abstract including 182 patients from eight countries [75].

The diabetogenic effects of corticosteroids and calcineurin inhibitors are the main causes of PTDM, a prevalent metabolic complication in KTRs affecting 10–20% of patients. Because PTDM increases the risk of infections, cardiovascular events, and graft malfunction, effective management is essential. Recent studies have indicated that SGLT2is can achieve effective glycemic and metabolic control in KTRs, as evidenced by reductions in HbA1c by 0.7–0.9%, weight loss of 3.2–3.4 kg, and improved lipid profiles without inducing hypoglycemia, substantial drug interactions with immunosuppressive medications, or adverse effects on allograft function. However, SGLT2i use in KTR patients may cause an acute temporary decline in eGFR, thought to be associated with SGLT2i-induced afferent arteriolar vasoconstriction [75, 76].

Administration of SGLT2is in KTRs has led to a 25% reduction in albuminuria, stabilization of eGFR, significant improvements in blood pressure control, and a decreased need for diuretics over 18 months. The putative mechanism by which SGLT2is protect transplanted kidneys from chronic allograft dysfunction is their capacity to lower intraglomerular pressure and proteinuria. Larger trials are needed to confirm the renoprotective advantages of SGLT2is in transplant populations, despite encouraging initial findings [6, 77].

### Safety considerations

The safety of SGLT2is in KTRs has been extensively studied, revealing manageable adverse effects with careful patient selection. Increased glycosuria may predispose patients to urinary and genital infections, although rates are comparable to those in the nontransplant population. Urinary tract infection (UTI) was the most frequent adverse effect [77]. While rare, euglycemic diabetic ketoacidosis can occur, particularly in cases of dehydration or abrupt insulin withdrawal. SGLT2is can also cause osmotic diuresis, potentially leading to volume depletion and hypotension, which is especially concerning for KTRs on multiple medications affecting fluid and electrolyte balance; hence, caution is advised for those on high-dose diuretics or with preexisting hypotension. Additionally, careful monitoring is essential to prevent electrolyte imbalances, particularly concerning potassium and magnesium levels. For stable KTRs, SGLT2is are usually safe and can be appropriately monitored [6].

Several ongoing trials seek to further elucidate the role of SGLT2is in KTRs. Additionally, in the DAPA-CKD and EMPA-KIDNEY trials [6, 78], preliminary findings revealed reductions in HbA1c, weight, and proteinuria, providing a foundation for assessing renoprotective and cardiovascular benefits.

Although further research is needed to fully understand the potential benefits of SGLT2is, there is currently inadequate data to recommend their use for nondiabetic KTRs [79].

### Recommendations by Kasralainy nephrology group (KANG)

- Due to the potential benefits, we advise kidney transplant recipients with diabetes (KTRs) to take SGLT2 inhibitors (SGLT2is). This recommendation is supported by emerging evidence from observational studies and small randomized controlled trials (RCTs). While large RCTs specifically in KTRs are limited, expert consensus also supports their use in this population, pending further large-scale RCTs.
- Hydration Counseling: Patients should be counseled to maintain proper hydration while on SGLT2is. This recommendation is based on expert consensus and physiological principles.
- Perineal Hygiene: Patients should be counseled on proper perineal hygiene to reduce the risk of urinary tract infections (UTIs).
- Avoidance in Recurrent UTI Cases: SGLT2is should be avoided in recipients with a history of recurrent UTIs. This recommendation is supported by observational studies and post-hoc analyses of RCTs. Expert consensus highlights the importance of UTI prevention in KTRs, who are already at higher risk for infections due to immunosuppression.

### SGLT2 inhibitors and polycystic kidney disease (PKD)

Autosomal dominant polycystic kidney disease (ADPKD) is the most common inherited kidney disease, characterized by the formation of numerous fluid-filled cysts primarily within the kidneys. It often progresses to end-stage kidney disease (ESKD) as the number and size of renal cysts increase [80].

Polycystic kidney disease (PKD) is associated with impaired autophagy, increased apoptosis, mitochondrial dysfunction, and altered metabolic processes, including altered glucose metabolism, increased glycolysis, and decreased oxidative phosphorylation [81].

SGLT2 inhibitors (SGLT2is) are currently established cardio- and renoprotective agents for both diabetic and nondiabetic conditions. Thus, ADPKD patients might theoretically benefit from this drug class in terms of kidney and/or heart protection. SGLT2is constitute a promising class for targeting energy metabolism and mitochondrial dysfunction. The mechanisms contributing to these benefits are still under investigation; however, the switch to a ketotic state (e.g., increased β-hydroxybutyrate) induced by glucosuria is likely the main mechanism. Moreover, SGLT2is have pleiotropic effects beyond blood glucose lowering, including anti-inflammatory effects that increase autophagy and improve mitochondrial function [82].

In addition to these metabolic effects, SGLT2is have been proposed to exert a beta-blocker-like effect on the kidneys, decreasing the energy demand of proximal tubule cells and possibly protecting them from a hypoxic environment [83].

Therefore, SGLT2 inhibitors decrease renal hypoxia, enhance nutrient deprivation signaling, suppress HIF-1α, and activate HIF-2α, which promotes erythrocytosis [84]. A recent review comparing the effects of SGLT2is and HIF-prolyl hydroxylase inhibitors on ADPKD suggested that SGLT2is might provide a better alternative to tolvaptan rather than HIF-prolyl hydroxylase inhibitors for patients with ADPKD [85]. Furthermore, in ADPKD, higher baseline albuminuria was associated with faster eGFR loss [86]. This rationale supports the potential benefit of SGLT2is in ADPKD.

The relationship between sodium-glucose cotransporter-2 (SGLT2) inhibitors and PKD has been explored in preclinical studies, but clinical evidence remains limited. A study by Kapoor et al. investigated the effects of the SGLT2 inhibitor dapagliflozin (DAPA) in a rat model of PKD. The study found that DAPA treatment led to increased glucose excretion and urine output but also resulted in hyperfiltration, albuminuria, and an increase in cyst volume and kidney weight in PCK rats. These findings suggest that while SGLT2 inhibitors may exhibit renoprotective effects in other contexts, their impact on PKD progression appears complex and potentially adverse [87, 88].

Published clinical trials with SGLT2is and ADPKD remain limited or nonexistent, as patients with ADPKD were excluded from clinical trials assessing kidney protection as the primary outcome in patients with or without diabetic CKD. Currently, there are two ongoing clinical trials on the use of SGLT2is in PKD patients. One trial (NCT05510115) is a randomized, double-blind, placebo-controlled trial involving 50 ADPKD patients with an eGFR of 30–90 mL/min/1.73 m². Patients receive empagliflozin or placebo over 12 months, with the primary outcome being the safety and tolerability of empagliflozin. The results are expected in 2026 [89]. The second study, EMPA-PKD (NCT06391450), is a double-blind RCT of empagliflozin versus placebo, enrolling 44 patients with PKD and following them for 18 months. The primary outcome is the change in total kidney volume before and after the study. The results of this trial are expected by the end of 2027 [90].

### Safety considerations

Many concerns regarding the use of SGLT2 inhibitors in PKD patients have been raised. The most important concern is the stimulation of vasopressin release secondary to SGLT2 inhibitor-induced osmotic diuresis, which could worsen rather than improve cystic disease. Another safety issue associated with SGLT2is in ADPKD is their potential to exacerbate hypovolemia, hypernatremia, and acute kidney injury when combined with tolvaptan, which prevents vasopressin-mediated water reabsorption.

Another issue is the risk of genitourinary infection with the use of SGLT2 inhibitors. An increasing number of infections affecting kidney cysts may have potentially severe consequences, including faster disease progression.

### Recommendations by Kasralainy nephrology group (KANG)

Trials to assess the feasibility of SGLT2 inhibitors in ADPKD are just beginning. Therefore, until further evidence is available, SGLT2 inhibitors should not be used in ADPKD patients outside of clinical trials. These recommendations align with the conclusions of the few available published preclinical trials [87, 88].

**Table 2:** Various renal effects of SGLT2 inhibitors

Renal Effect	Mechanism
Reduction in glomerular hyperfiltration	Afferent arteriolar vasoconstriction reduces intraglomerular pressure, slowing the progression of CKD by reducing hyperfiltration-related damage through activation of tubuloglomerular feedback.
Slowed eGFR decline	Lowered glomerular pressure and reduced inflammation.
Reduced risk of end-stage kidney disease (ESKD)	Renoprotection through decreased tubular stress and improved hemodynamics.
Cardiorenal protection	Improved glycemic control, blood pressure, and uric acid levels
Potential risk of acute kidney injury (AKI)	Volume depletion due to diuresis

### Limitations of the study

This review is limited by the small sample sizes and short follow-up durations in many of the included studies, which complicates the evaluation of the long-term efficacy and safety of SGLT2 inhibitors in kidney diseases. Additionally, the majority of studies have focused primarily on diabetic kidney disease with proteinuria, with insufficient data on non-diabetic conditions such as autosomal dominant polycystic kidney disease (ADPKD), renal anemia, renal bone disease, and lupus nephritis, which limits the generalizability of the findings. The use of various study designs, including observational and retrospective analyses, introduces potential biases, thereby affecting the robustness of the conclusions. Moreover, the paucity of data from low- and middle-income countries further restricts the applicability of these findings across diverse populations.

## Conclusion

SGLT2 inhibitors exhibit dual effects on kidney disease progression, with potential risks of adverse renal outcomes as well as benefits of favorable prognostic improvements (Table 2). Consequently, risk-benefit stratification is essential before initiating these agents to optimize therapeutic outcomes and mitigate serious complications. The Kasralainy Nephrology Group (KANG) clinical recommendations present a structured framework for assessing kidney disease patients across diverse clinical phenotypes, guiding the use of SGLT2 inhibitors in conditions commonly encountered in nephrology practice (Fig. 1) (Table 3). Further research is necessary to resolve existing controversies in the literature regarding the effects of these agents on comorbid conditions in kidney disease patients.

**Table 3:** Recommendations on the use of SGLT2 inhibitors in different kidney diseases

Category	Who Should Receive SGLT2 Inhibitors	Who Should Avoid SGLT2 Inhibitors	Monitor Recommendation
**SGLT2 inhibitors and chronic kidney disease (CKD)**	- Patients with hypertension, especially if accompanied by proteinuria - Patients with diabetic kidney disease (DKD) to reduce progression of kidney disease and cardiovascular risk - Non-diabetic CKD patients with albuminuria (e.g., UACR > 30 mg/g) or eGFR ≥ 20 mL/min/1.73 m²	- Patients with eGFR < 20 mL/min/1.73 m² (limited efficacy) - Severe volume depletion or hypotension - Patients with heart failure if EF < 50% - Patients with recurrent UTI, recurrent genital infections, patients with renal stones - Children, pregnancy, and elderly	- Monitor eGFR and albuminuria regularly to assess efficacy - Check for volume depletion and adjust diuretics as needed
**SGLT2 inhibitors and dialysis**	Limited evidence; in selected patients not yet on dialysis but approaching ESKD to reduce cardiovascular events	Patients already on dialysis (excluded from most trials)	Monitor for volume status and avoid dehydration in advanced CKD stages
**SGLT2 inhibitors and acute kidney injury (AKI)**	- Prevention of AKI in CKD patients (strong evidence) - Prevention of contrast-induced nephropathy (weak evidence)	Patients with active AKI or hemodynamic instability (e.g., sepsis, volume depletion)	- Monitor renal function closely after initiating therapy - To watch for contrast-induced nephropathy: serum creatinine, urea, urine output monitoring every 12 h
**SGLT2 inhibitors and bone mineral disease**	- T2DM patients without high fracture risk, prioritizing cardiovascular/renal benefits	- High fracture risk (osteoporosis, prior fracture, CKD-MBD) - Active foot disease (infection/ulceration)	Monitor CKD-MBD markers (calcium, phosphate, PTH) in CKD
**SGLT2 inhibitors and renal anemia**	Mild and moderate anemia, mild & moderate anemia with heart failure	Severe anemia, Hb range 10–12 on ESAs, anemic patients receiving IV iron	Regular hemoglobin and hematocrit checks to avoid erythrocytosis
**SGLT2 inhibitors and glomerulonephritis**	- Potential use in diabetic glomerulopathy or proteinuric glomerular diseases (e.g., with IgA nephropathy and focal segmental glomerulosclerosis) to reduce albuminuria and CKD progression - Patients with hyperkalemia risk, especially when combined with renin-angiotensin system (RAS) inhibitors	Non-proteinuric glomerulonephritis without clear benefit demonstrated by trials	- Regular urine albumin-creatinine ratio (UACR) monitoring to assess response to therapy - eGFR to detect any acute declines - Serum potassium: monitoring especially in patients on RAS inhibitors or mineralocorticoid receptor antagonists (MRA)
**SGLT2 inhibitors and kidney transplantation**	Diabetic kidney transplant recipients for glycemic control and potential cardiorenal benefits	Early post-transplant period (< 12 months); risk of infections or graft instability is higher; high risk of volume depletion or acute kidney injury (AKI)	Monitor renal graft function, blood glucose, and infection risk periodically
**SGLT2 inhibitors and polycystic kidney disease (PKD)**	None	All patients with PKD	None applicable

## Data Availability

No datasets were generated or analysed during the current study.

## Abbreviations

ADPKD: Autosomal dominant polycystic kidney disease
AKI: Acute kidney injury
BMD: Bone mineral disease
CKD: Chronic kidney disease
CVOTs: Cardiovascular outcome trials
DKD: Diabetic kidney disease
eGFR: Estimated glomerular filtration rate
EPO: Erythropoietin
ESKD: End-stage kidney disease
FDAERS: Food and Drug Administration Adverse Event Report System
GLP-1: Glucagon-Like Peptide-1 Agonists
HFpEF: Heart failure with preserved ejection fraction
HFrEF: Heart failure with reduced ejection fraction
KANG: Kasralainy Nephrology Group
KTRs: Kidney transplant recipients
MeSH: Medical Subject Headings
MRAs: Mineralocorticoid Receptor Antagonists
NRP: Nephrotic-range proteinuria
PKD: Polycystic kidney disease
PTDM: Posttransplant diabetes mellitus
RAS: Renin‒angiotensin system
SGLT2i: Sodium-glucose cotransporter 2 inhibitors
T2DM: Type 2 diabetes mellitus
UTIs: Urinary tract infections

## References

[1] Packer M, Wilcox CS, Testani JM. Critical analysis of the effects of SGLT2 inhibitors on renal tubular sodium, water and chloride homeostasis and their role in influencing heart failure outcomes. Circulation. 2023;148(4). 10.1161/CIRCULATIONAHA.123.0643. 10.1161/CIRCULATIONAHA.123.064346 PMC10358443 37486998

[2] Zhou Y, Li C, Li H. Renal protection by sodium-glucose cotransporter 2 inhibitors and its underlying mechanisms in diabetic kidney disease. J Diabetes Complicat. 2018;32(7):720–5. 10.1016/j.jdiacomp.2018.03.008. 10.1016/j.jdiacomp.2018.04.011 29880432

[3] Mima A. Sodium-Glucose cotransporter 2 inhibitors in patients with Non-Diabetic chronic kidney disease. Adv Therapy. 2021;38(5):2201–12. 10.1007/s12325-021-01735-5. 10.1007/s12325-021-01735-5 33860925

[4] Mima A. Mitochondria-targeted drugs for diabetic kidney disease. Heliyon. 2022;8(2):e08878. 10.1016/j.heliyon.2022.e08878. 35265754 10.1016/j.heliyon.2022.e08878 PMC8899696

[5] Koh ES, Kim G-H, Chung S. Intrarenal mechanisms of sodium-glucose cotransporter-2 inhibitors on tubuloglomerular feedback and natriuresis. Endocrinol Metabolism. 2023;38(4):359–72. 10.3803/EnM.2023.1764. 10.3803/EnM.2023.1764 PMC10475968 37482684

[6] Heerspink HJL, et al. Dapagliflozin in patients with chronic kidney disease. N Engl J Med. 2020;383(15):1436–46. 32970396 10.1056/NEJMoa2024816

[7] Packer M, et al. Cardiovascular and renal outcomes with empagliflozin in heart failure. N Engl J Med. 2020;383(15):1413–24. 32865377 10.1056/NEJMoa2022190

[8] Yau K, Dharia A, Alrowiyti I, Cherney DZI. Prescribing SGLT2 inhibitors in patients with CKD: expanding indications and practical considerations. Kidney Int Rep. 2022;7(7):1463–76. 10.1016/j.ekir.2022.04.094. 35812300 10.1016/j.ekir.2022.04.094 PMC9263228

[9] Neuen BL, Young TP, Heerspink HJL, et al. Sodium-glucose cotransporter 2 inhibitors for chronic kidney disease: A systematic review and meta-analysis. Kidney Int. 2020;98(2):287–98. 10.1016/j.kint.2020.03.012.

[10] Hahr AJ, Molitch ME. Management of diabetes mellitus in patients with CKD: core curriculum 2022. Am J Kidney Dis. 2022;79:728–36. 10.1053/j.ajkd.2021.05.023. 34600745 10.1053/j.ajkd.2021.05.023

[11] Din SE, Salem UAA, M. M., Abdulazim DO. Sodium-glucose cotransporter 2 inhibitors as the first universal treatment of chronic kidney disease. Nefrologia. 2022;42:390–403. 10.1016/j.nefro.2021.03.014. 36460429 10.1016/j.nefroe.2022.08.001

[12] Mima A. A narrative review of diabetic kidney disease: previous and current Evidence-Based therapeutic approaches. Adv Therapy. 2022;39(8):3488–500. 10.1007/s12325-022-02223-0. 10.1007/s12325-022-02223-0 35751762

[13] Li N et al. (2022). Effects of SGLT2 inhibitors on cardiovascular outcomes in patients with stage 3/4 CKD: A meta-analysis. PLoS ONE, 17. 10.1371/journal.pone.0261986 PMC8754287 35020750

[14] Bailey CJ, Day C, Bellary S. Renal protection with SGLT2 inhibitors: effects in acute and chronic kidney disease. Curr Diab Rep. 2022;22:39–52. 10.1007/s11892-021-01442-z. 35113333 10.1007/s11892-021-01442-z PMC8888485

[15] Perkovic V, et al. Canagliflozin and renal outcomes in type 2 diabetes and nephropathy. N Engl J Med. 2019;380:2295–306. 30990260 10.1056/NEJMoa1811744

[16] Bellary S, Barnett AH. SGLT2 inhibitors in older adults: overcoming the age barrier. Lancet Healthy Longev. 2023;4:e127–8. 10.1016/S2666-7568(23)00039-9. 37003269 10.1016/S2666-7568(23)00039-9

[17] Kitaoka K et al. (2024). Kidney outcomes of SGLT2 inhibitors among older patients with diabetic kidney disease in real-world clinical practice: the Japan chronic kidney disease database ex. BMJ Open Diabetes Res Care, 12. 10.1136/bmjdrc-2024-004115 PMC11141184 38816204

[18] Gross O, Haffner D, Schaefer F, Weber LT. SGLT2 inhibitors: approved for adults and cats but not for children with CKD. Nephrol Dialysis Transplantation. 2024;39:907–9. 10.1093/ndt/gfae029. 10.1093/ndt/gfae029 38308509

[19] Wisbaum A, Gaudreau S, Cloutier I, Robert P, Kolment R, Beauchesne MF, Couture J. Real-Time use of SGLT2i verified in predialysis: the RSVP Cross-sectional study. Ann Pharmacother. 2024;8:10600280241245995. 10.1177/10600280241245995 PMC11566075 38736313

[20] Oliva-Damaso N, Delanaye P, Oliva-Damaso E, Payan J, Glassock RJ. Risk-based versus GFR threshold criteria for nephrology referral in chronic kidney disease. Clin Kidney J. 2022;15(11):1996–2005. 36325015 10.1093/ckj/sfac104 PMC9613424

[21] D’Andrea E, Wexler DJ, Kim SC, Paik JM, Alt E, Patorno E. Comparing effectiveness and safety of SGLT2 inhibitors vs DPP-4 inhibitors in patients with type 2 diabetes and varying baseline HbA1c levels. JAMA Intern Med. 2023;183(3):242–54. 36745425 10.1001/jamainternmed.2022.6664 PMC9989905

[22] van der Aart AB, de Boer RA, Heerspink HJ. Kidney and heart failure outcomes associated with SGLT2 inhibitor use. Nat Rev Nephrol. 2022;18(5):294–306. 35145275 10.1038/s41581-022-00535-6

[23] Maruyama-Sakurai K, Tachimori H, Saito E, Kohsaka S, Segawa Y, Miyata H, Igarashi A. Cost‐effectiveness of sodium‐glucose cotransporter‐2 inhibitors in the treatment of diabetic nephropathy in Japan. Diabetes Obes Metabolism. 2024;26(12):5546–55. 10.1111/dom.15832 39344831

[24] Klein KR, Lingvay I, Tuttle KR, Flythe JE. Glycemic management and individualized diabetes care in Dialysis-Dependent kidney failure. Diabetes Care. 2025;48(4):728–36. 39693267 10.2337/dci24-0081 PMC11770169

[25] Huang B, Yen CL, Wu CY, Tsai CY, Chen JJ, Hsiao CC, Chen YC, Hsieh IC, Yang HY. SGLT2 inhibitors reduce the risk of renal failure in CKD stage 5 patients with type 2 DM. Sci Rep. 2025;15(1):5872. 39966427 10.1038/s41598-024-81973-z PMC11836049

[26] Hammad H, Shaaban A, Philips MV, Fayed A, Abdelaziz TS. Effect of sodium-glucose transporter 2 inhibitor empagliflozin on proteinuria and kidney function progression in patients with nondiabetic glomerulonephritis: a pilot superiority randomized controlled trial. Int Urol Nephrol. 2023;55:2321–6. 36872420 10.1007/s11255-023-03539-8 PMC10406661

[27] Sridhar VS, Tuttle KR, Cherney DZI. We can finally stop worrying about SGLT2 inhibitors and acute kidney injury. Am J Kidney Dis. 2020;76(4):454–6. 32712014 10.1053/j.ajkd.2020.05.014

[28] Chung M-C, Hung P-H, Hsiao P-J, Wu L-Y, Chang C-H, Hsiao K-Y, et al. Sodium-Glucose transport protein 2 inhibitor use for type 2 diabetes and the incidence of acute kidney injury in Taiwan. JAMA Netw Open. 2023;6(3):e230453–230453. 36811856 10.1001/jamanetworkopen.2023.0453 PMC9947724

[29] Neuen BL, Young T, Heerspink HJL, Neal B, Perkovic V, Billot L, et al. SGLT2 inhibitors for the prevention of kidney failure in patients with type 2 diabetes: a systematic review and meta-analysis. Lancet Diabetes Endocrinol. 2019;7(11):845–54. 31495651 10.1016/S2213-8587(19)30256-6

[30] Menne J, Dumann E, Haller H, Schmidt BMW. (2019). Acute kidney injury and adverse renal events in patients receiving SGLT2-inhibitors: A systematic review and meta-analysis. PLoS Med, 16(12), e1002983. 10.1371/journal.pmed.1002983 PMC6901179 31815931

[31] van Bommel EJM, Muskiet MHA, Tonneijck L, Kramer MHH, Nieuwdorp M, van Raalte DH. SGLT2 Inhibition in the diabetic Kidney—From mechanisms to clinical outcome. Clin J Am Soc Nephrol. 2017;12(4):700–7. 28254770 10.2215/CJN.06080616 PMC5383382

[32] Yao D, Wang S, Wang M, Lu W. Renoprotection of Dapagliflozin in human renal proximal tubular cells via the Inhibition of the high mobility group box 1-receptor for advanced glycation end products-nuclear factor-κB signaling pathway. Mol Med Rep. 2018;18(1):863–72. 10.3892/mmr.2018.9393 30132524

[33] Laursen JC, Søndergaard-Heinrich N, de Melo JML, Haddock B, Rasmussen IKB, Safavimanesh F, et al. Acute effects of Dapagliflozin on renal oxygenation and perfusion in type 1 diabetes with albuminuria: A randomized, double-blind, placebo-controlled crossover trial. EClinicalMedicine. 2021;37:100895. 34386735 10.1016/j.eclinm.2021.100895 PMC8343250

[34] Maayah ZH, Ferdaoussi M, Takahara S, Soni S, Dyck JRB. Empagliflozin suppresses inflammation and protects against acute septic renal injury. Inflammopharmacology. 2021;29(1):269–79. 32564182 10.1007/s10787-020-00732-4

[35] Huang X, Guo X, Yan G, Zhang Y, Yao Y, Qiao Y, et al. Dapagliflozin attenuates Contrast-induced acute kidney injury by regulating the HIF-1α/HE4/NF-κB pathway. J Cardiovasc Pharmacol. 2022;79(6):863–72. 10.1097/FJC.0000000000001268 PMC9162274 35383661

[36] Meregildo-Rodriguez E, Asmat-Rubio M, Vásquez-Tirado GA. (2023). SGLT-2 inhibitors and prevention of contrast-induced nephropathy in patients with diabetes undergoing coronary angiography and percutaneous coronary interventions: systematic review and meta-analysis. Front Endocrinol, 14. 10.3389/fendo.2023.1307715 PMC10765513 38179307

[37] Cai D, Chen Q, Mao L, Xiao T, Wang Y, Gu Q, Wang Q, Ji Y, Sun L. Association of SGLT2 inhibitor Dapagliflozin with risks of acute kidney injury and all-cause mortality in acute myocardial infarction patients. Eur J Clin Pharmacol. 2024;80(4):613–20. 38319348 10.1007/s00228-024-03623-7 PMC10937750

[38] Rossing P, Caramori ML, Chan JCN, Heerspink HJL, Hurst C, Khunti K, et al. Executive summary of the KDIGO 2022 clinical practice guideline for diabetes management in chronic kidney disease: an update based on rapidly emerging new evidence. Kidney Int. 2022;102(5):990–9. 36272755 10.1016/j.kint.2022.06.013

[39] Kahn SE, et al. Effects of SGLT-2 Inhibition on glucose homeostasis and bone metabolism: mechanistic insights. Diabetes Care. 2022;45(12):e171–9.36205432

[40] Zhang Y, Xu L. Impact of SGLT2 inhibitors on bone mineral density in patients with type 2 diabetes: A systematic review and Meta-analysis. Diabetes Therapy. 2021;12(5):1245–56.

[41] Nath S, Choudhury M. SGLT2 inhibitors and bone health: A comprehensive review. J Endocrinol Metabolism. 2022;12(4):245–53.

[42] Gerard SC, Hentz JG. Long-term effects of SGLT2 inhibitors on bone health: risk of fracture and implications for clinical practice. Metabolism Reviews. 2023;64(5):288–300.

[43] Gersing AS, et al. Sodium-Glucose cotransporter 2 inhibitors and bone mineral density: A systematic review and Meta-Analysis. J Bone Miner Res. 2020;35(9):1699–709.

[44] Zinman B, et al. Empagliflozin, cardiovascular outcomes, and mortality in type 2 diabetes. N Engl J Med. 2015;373(22):2117–28. 26378978 10.1056/NEJMoa1504720

[45] Comprehensive Medical Evaluation and Assessment of Comorbidities. Standards of care in Diabetes—2025. Diabetes Care, 48(Supplement_1), S59–85. 10.2337/dc25-S004 PMC11635044 39651988

[46] Danish Society for Diabetes (DSD) & European Association for the Study of Diabetes (EASD). 2023 EASD/ADA consensus report on SGLT2 inhibitors. Diabetes Care. 2023;46(9):1714–24.

[47] Wang X, Zhang F, Zhang Y, et al. Effect of SGLT2 inhibitors on fractures, BMD, and bone metabolism markers in patients with type 2 diabetes mellitus: A systematic review and Meta-Analysis. Osteoporos Int. 2023;34(12):2013–25. 37695339 10.1007/s00198-023-06908-2

[48] Li X, Li T, Cheng Y et al. (2019). Effects of SGLT2 inhibitors on fractures and bone mineral density in type 2 diabetes: an updated Meta-Analysis. Diab/Metab Res Rev, 35(7), e3170. 10.1002/dmrr.3170 30983141

[49] Kaze AD, Patorno E, Paik JM. Safety of SGLT2i with regard to bone and mineral metabolism in patients with CKD. Curr Opin Nephrol Hypertens. 2023;32(4):324–9. 37195239 10.1097/MNH.0000000000000887

[50] Kanbay M, Tapoi L, Ureche C, Tanriover C, Cevik E, Demiray A, et al. Effect of sodium-glucose cotransporter 2 inhibitors on hemoglobin and hematocrit levels in type 2 diabetes: a systematic review and meta-analysis. Int Urol Nephrol. 2022;54(4):827–41. 34273060 10.1007/s11255-021-02943-2

[51] Ferreira JP, Anker SD, Butler J, Filippatos G, Iwata T, Salsali A, et al. Impact of anemia and the effect of empagliflozin in heart failure with reduced ejection fraction: findings from EMPEROR-Reduced. Eur J Heart Fail. 2022;24(4):708–15. 34957660 10.1002/ejhf.2409 PMC9303456

[52] Koshino A, Schechter M, Chertow GM, Vart P, Jongs N, Toto RD et al. (2023). Dapagliflozin and anemia in patients with chronic kidney disease. NEJM Evid, 2. 10.1056/EVIDoa2300049 38320128

[53] Fuchs Andersen C, Omar M, Glenthøj A, El Fassi D, Møller HJ, Lindholm Kurtzhals JA, et al. Effects of empagliflozin on erythropoiesis in heart failure: data from the empire HF trial. Eur J Heart Fail. 2023;25(5):226–34. 36377106 10.1002/ejhf.2735

[54] Stefánsson BV, Heerspink HJL, Wheeler DC, Sjöström CD, Greasley PJ, Sartipy P, et al. Correction of anemia by Dapagliflozin in patients with type 2 diabetes. Journal of Diabetes and its Complications; 2020. p. 34. 10.1016/j.jdiacomp.2020.107729 32948397

[55] Cases A, Cigarrán S, Górriz JL, Nuñez J. Effect of SGLT2 inhibitors on anemia and their possible clinical implications. Nefrologia. 2024;44(2):119–31. 38604895 10.1016/j.nefroe.2024.03.011

[56] Sano M, Goto S. Possible mechanism of hematocrit elevation by sodium-glucose cotransporter 2 inhibitors and associated beneficial renal and cardiovascular effects. Circulation. 2019;139:1985–7. 31009585 10.1161/CIRCULATIONAHA.118.038881

[57] Mayer B, Németh K, Krepuska M, Myneni VD, Maric D, Tisdale JF et al. (2017). Vasopressin stimulates the proliferation and differentiation of red blood cell precursors and improves recovery from anemia. Sci Transl Med, 9. 10.1126/scitranslmed.aao1632 PMC6309406 29187641

[58] Eickhoff MK, Dekkers CCJ, Kramers BJ, Laverman GD, Frimodt-Møller M, Jørgensen NR et al. (2019). Effects of Dapagliflozin on volume status when added to renin-angiotensin system inhibitors. J Clin Med, 8. 10.3390/jcm8060779 PMC6616433 31159350

[59] Packer M. Critical reanalysis of the mechanisms underlying the cardiorenal benefits of SGLT2 inhibitors and reaffirmation of the nutrient deprivation signaling/autophagy hypothesis. Circulation. 2022;146:1383–405. 36315602 10.1161/CIRCULATIONAHA.122.061732 PMC9624240

[60] Ghanim H, Abuaysheh S, Hejna J, Green K, Batra M, Makdissi A et al. (2020). Dapagliflozin suppresses Hepcidin and increases erythropoiesis. J Clin Endocrinol Metab, 105. 10.1210/clinem/dgaa057 32044999

[61] Fitchett D, Inzucchi SE, Zinman B, Wanner C, Schumacher M, Schmoor C, et al. Mediators of the improvement in heart failure outcomes with empagliflozin in the EMPA-REG OUTCOME trial. ESC Heart Fail. 2021;8:4517–27. 34605192 10.1002/ehf2.13615 PMC8712833

[62] Lorenzo M, Miñana G, Palau P, Amiguet M, Seller J, Garcia Pinilla JM, et al. Short-term changes in hemoglobin and changes in functional status, quality of life and natriuretic peptides after Dapagliflozin in heart failure with reduced ejection fraction. Results of the DAPA-VO2 study. ESC Heart Fail. 2023;10(1):503–13. 10.1016/j.cardfail.2023.02.008 36871614

[63] Palmer SC, Navaneethan SD, Craig JC, Johnson DW, Tonelli M, Garg AX, et al. Meta-analysis: erythropoiesis-stimulating agents in patients with chronic kidney disease. Ann Intern Med. 2010;153(1):23–33. 10.7326/0003-4819-153-1-201007060-00252. 20439566 10.7326/0003-4819-153-1-201007060-00252

[64] Ku E, Del Vecchio L, Eckardt KU, Haase VH, Johansen KL, Nangaku M et al. for Conference Participants. Novel anemia therapies in chronic kidney disease: conclusions from a kidney disease: improving global outcomes (KDIGO) controversies conference. Kidney International, 104(4), 655–680 (2023). 10.1016/j.kint.2023.05.009 10.1016/j.kint.2023.05.009 37236424

[65] Swedberg K, Young JB, Anand IS, Cheng S, Desai AS, Diaz R, et al. RED-HF committees; RED-HF investigators. Treatment of anemia with Darbepoetin Alfa in systolic heart failure. N Engl J Med. 2013;368(13):1210–9. 10.1056/NEJMoa1214865. 23473338 10.1056/NEJMoa1214865

[66] Imprialos KP, Boutari C, Stavropoulos K, Doumas M, Karagiannis AI. Stroke paradox with SGLT-2 inhibitors: a play of chance or a viscosity-mediated reality? J Neurol Neurosurg Psychiatry. 2017;88(3):249–53. 10.1136/jnnp-2016-314704. 27895093 10.1136/jnnp-2016-314704

[67] Koshino A, et al. Dapagliflozin and Anemia in patients with chronic kidney disease. NEJM Evid. 2023;2(12). 10.1056/EVIDoa2300113. 10.1056/EVIDoa2300049 38320128

[68] Oshima M, et al. Effects of Canagliflozin on anemia in patients with type 2 diabetes and chronic kidney disease: a post-hoc analysis from the CREDENCE trial. Lancet Diabetes Endocrinol. 2020;8(11):903–14. 33065060 10.1016/S2213-8587(20)30300-4

[69] Locatelli F, Del Vecchio L. Cardio-renoprotective effects of SGLT2 inhibitors—the role of anemia correction. Nephrol Dialysis Transplantation. 2024;39(6):904–6. 10.1093/ndt/gfae019 38263523

[70] Caravaca-Fontán F, Stevens K, Padrón M, Huerta A, Montomoli M, Villa J, González F, Vega C, López Mendoza M, Fernández L, Shabaka A. Sodium-glucose cotransporter 2 Inhibition in primary and secondary glomerulonephritis. Nephrol Dialysis Transplantation. 2024;39(2):328–40. 10.1093/ndt/gfad175 37550217

[71] Hu G, Wu Y, Chen F, Tang J. Progress of SGLT2 inhibitors in the treatment of common immune-related nephropathies. Int Urol Nephrol. 2024;56(9):1–7. 38963512 10.1007/s11255-024-04141-2

[72] Elkeraie A, Zyada R, Elrggal ME, Elrggal M. Safety of SGLT2 inhibitors in patients with different glomerular diseases treated with immunosuppressive therapies. Eur J Clin Pharmacol. 2023;79(7):961–6. 37199747 10.1007/s00228-023-03508-1

[73] Ahmed RM, Rakha NK, Yousry A, Soliman AR. Potential impact of sodium-glucose cotransporter (SGLT2) inhibitors on cholesterol fractions in stage 3 chronic kidney disease. Egypt J Intern Med. 2024;36(1):83.

[74] Ramakrishnan P, Garg N, Pabich S, Mandelbrot DA, Swanson KJ. Sodium-glucose cotransporter-2 inhibitor use in kidney transplant recipients. World J Transplantation. 2023;13(5):239. 10.5500/wjt.v13.i5.239 PMC10514750 37746038

[75] Ujjawal A, Schreiber B, Verma A. Sodium-glucose cotransporter-2 inhibitors (SGLT2i) in kidney transplant recipients: what is the evidence? Therapeutic Adv Endocrinol Metabolism. 2022;13:20420188221090001. 10.1177/20420188221090001 PMC9016587 35450095

[76] Halden TA, Kvitne KE, Midtvedt K, Rajakumar L, Robertsen I, Brox J, Bollerslev J, Hartmann A, Åsberg A, Jenssen T. Efficacy and safety of empagliflozin in renal transplant recipients with posttransplant diabetes mellitus. Diabetes Care. 2019;42(6):1067–74. 30862658 10.2337/dc19-0093

[77] Sánchez Fructuoso AI, Raba B, Banegas Deras A, Vigara Sánchez E, San Cecilio LAV, Franco Esteve R, Cruzado Vega A, Gavela L, Martínez E, González Garcia ME, Saurdy Coronado P, Morales N. D. Sodium-glucose cotransporter-2 inhibitor therapy in kidney transplant patients with type 2 or posttransplant diabetes: an observational multicenter study. Clin Kidney J. 2023;16(6):1022–34. 37260993 10.1093/ckj/sfad007 PMC10229265

[78] EMPA-Kidney Collaborative Group. Empagliflozin in patients with chronic kidney disease. N Engl J Med. 2023;388(2):117–27. 36331190 10.1056/NEJMoa2204233 PMC7614055

[79] Polychronopoulou E, Bourdon F, Teta D. SGLT2 inhibitors in diabetic and nondiabetic kidney transplant recipients: current knowledge and expectations. Front Nephrol. 2024;4:1332397. 38685973 10.3389/fneph.2024.1332397 PMC11056593

[80] Jdiaa SS, Mustafa RA, Yu ASL. Treatment of Autosomal-Dominant polycystic kidney disease. Am J Kidney Dis. 2024;84(1):185–94. 10.1053/j.ajkd.2024.08.008 39424253

[81] Nowak KL, Hopp K. Metabolic reprogramming in autosomal dominant polycystic kidney disease: evidence and therapeutic potential. Clin J Am Soc Nephrol. 2020;15(4):577–84. 32086281 10.2215/CJN.13291019 PMC7133124

[82] Sen T, Heerspink HJ. L. A kidney perspective on the mechanism of action of sodium-glucose cotransporter 2 inhibitors. Cell Metabol. 2021;33(4):732–9. 10.1016/j.cmet.2021.02.016 33691091

[83] Fernandez-Fernandez B, Sarafidis P, Kanbay M, et al. SGLT2 inhibitors for nondiabetic kidney disease: drugs to treat CKD that also improve glycemia. Clin Kidney J. 2020;13(5):728–33. 33123352 10.1093/ckj/sfaa198 PMC7577767

[84] Packer M. Mechanisms leading to differential hypoxia-inducible factor signaling in the diabetic kidney: modulation by SGLT2 inhibitors and hypoxia mimetics. Am J Kidney Dis. 2021;77(2):280–6. 32711072 10.1053/j.ajkd.2020.04.016

[85] Patel DM, Dahl NK. Examining the role of novel CKD therapies for ADPKD patients. Kidney360, 2(7), 1036–41 (2021). 10.34067/KID.0007422020 PMC8791369 35373079

[86] Gansevoort RT, Meijer E, Chapman AB, et al. Albuminuria and Tolvaptan in autosomal-dominant polycystic kidney disease: results of the TEMPO 3:4 trial. Nephrol Dialysis Transplantation. 2016;31(11):1887–94. 10.1093/ndt/gfv422 PMC6367945 26681730

[87] Afsar B, Afsar RE, Demiray A, Altay S, Korkmaz H, Yildiz A, Covic A, Ortiz A, Kanbay M. Sodium-glucose cotransporter Inhibition in polycystic kidney disease: fact or fiction. Clin Kidney J. 2022;15(7):1275–83. 35756735 10.1093/ckj/sfac029 PMC9217633

[88] Kapoor S, Rodriguez D, Riwanto M et al. Effect of Sodium-Glucose cotransport Inhibition on polycystic kidney disease progression in PCK rats. PLoS ONE, 10(4), e0125603 (2015). 10.1371/journal.pone.0125603 PMC4416041 25927597

[89] ClinicalTrials.gov. NCT05510115, feasibility-of-study-of-empagliflozin-in patients-with-autosomal-dominant-polycystic-kidney-disease 10.1159/000549447 PMC12854092 41505371

[90] ClinicalTrials.gov. NCT06391450. Study of empagliflozin in patients with autosomal dominant polycystic kidney disease (EMPA-PKD). 10.1136/bmjopen-2024-088317 PMC11647394 39675824

